# Emergency Physician-Performed Point-of-Care Ultrasound of a Renal Mass

**DOI:** 10.7759/cureus.48547

**Published:** 2023-11-09

**Authors:** Annie Au, Justin Harris, Gabriel Cabrera Correa, Eric J Kalivoda

**Affiliations:** 1 Emergency Medicine, Hospital Corporation of America (HCA) Healthcare/University of South Florida (USF) Morsani College of Medicine Graduate Medical Education (GME) Consortium, Brandon, USA

**Keywords:** emergency medicine ultrasound, incidental radiological findings, renal mass, ultrasonography, point-of-care ultrasound

## Abstract

Emergency physicians (EPs) frequently integrate point-of-care ultrasound (POCUS) into the initial bedside evaluation of patients presenting to the emergency department with acute flank pain. A POCUS-first diagnostic approach can allow EPs to promptly assess for life-threatening pathologies of the aorta and gallbladder. POCUS is also a critical bedside tool to determine renal causes of acute flank pain, such as hydronephrosis in the setting of nephrolithiasis, subcapsular hematomas, renal abscesses, pyelonephritis, and renal masses. This report illustrates a case in which EP-performed POCUS led to the incidental diagnosis of a malignant renal mass in a patient presenting with flank pain. We review the specifics of the ultrasound, computed tomography (CT), and magnetic resonance imaging (MRI) Bosniak classification system used by radiologists for risk stratification of cystic renal masses (CRMs).

## Introduction

The emergency department (ED) evaluation of flank pain typically incorporates an imaging modality to determine the underlying etiology. The initial diagnostic choice with either ultrasonography (US) or computed tomography (CT) largely depends on the clinical scenario. Ultrasound-first approaches for the emergency physician (EP) have been proposed, particularly in suspected cases of uncomplicated renal colic, with the purpose of decreasing lifetime radiation exposure without incurring serious adverse events, return ED visits, or hospitalizations [1-6]. Notably, CT imaging remains important for establishing an alternative diagnosis to nephrolithiasis in 10-15% of patients [1,2]. Point-of-care ultrasound (POCUS) is a vital bedside tool for EPs to rapidly discern other causes of acute flank pain such as aortic aneurysms, subcapsular hematomas, renal abscesses, pyelonephritis, renal masses, and biliary pathologies [1,4]. Previous reports have described EP-performed POCUS identification of renal masses as the etiology of flank pain [7,8].

Radiologists utilize the Bosniak classification system by CT and magnetic resonance imaging (MRI) for risk stratification of cystic renal masses (CRMs) [9,10]. Simple renal cysts (Bosniak I) are generally considered benign with the features of a well-defined homogeneous cystic mass and a smooth thin wall ≤ 2 mm. Conversely, more complex renal cysts (Bosniak IV) include representative features of an ill-defined heterogeneous cystic mass with an irregular thickened wall and/or septa ≥ 4 mm and enhancing nodules/protrusions with obtuse margins [9-11]. The change in morphology or progression of solid elements, rather than size and growth rate, are the more crucial factors during long-term surveillance of cystic renal masses [9]. Malignancy rates for Bosniak I and Bosniak IV are 3.2% and 91%, respectively; therefore, Bosniak category III-IV cystic masses typically warrant surgical treatment [9-11]. This case report highlights the role of EP-performed POCUS regarding the diagnosis and subsequent ED management of malignant renal masses.

## Case presentation

A 54-year-old female with a past medical history of hypertension, dyslipidemia, coronary artery disease, transient ischemic attack, venous thromboembolism, polycythemia vera, hiatal hernia repair, and tobacco use presented to the ED with one week of constant, mild severity, dull right flank pain with the associated decreased appetite. She denied abdominal pain otherwise. She denied back pain, fever, chills, nausea, vomiting, hematuria, dysuria, urinary frequency/urgency, diarrhea, constipation, dark or bloody stools, chest pain, palpitations, shortness of breath, syncope, or any other associated symptoms. The patient denied abdominal or flank trauma. Upon arrival, her vital signs were a temperature of 36.7°C, blood pressure of 124/86 mmHg, heart rate of 98 beats per minute, respiratory rate of 18 breaths per minute, and oxygen saturation of 97% on room air. On physical examination, she was well-appearing and in no acute distress, and reproducible tenderness of the right flank was appreciated. Abdominal quadrants were non-tender without rebound, guarding, or peritoneal signs, and costovertebral angle tenderness was not elicited.

**Figure 1:**
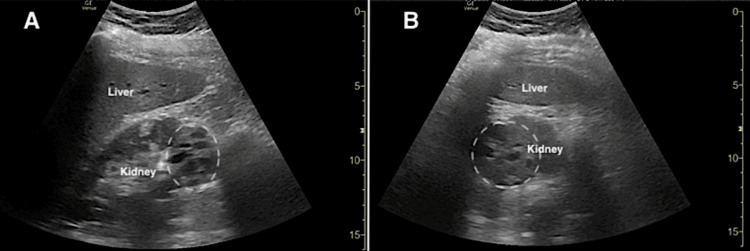
Point-of-care ultrasound demonstrating a renal mass (dotted circle) of the right kidney in longitudinal (A) and transverse (B) planes.

Emergency medicine resident physicians and an ultrasound fellowship-trained attending EP subsequently performed POCUS to assess for hydronephrosis in the clinical setting of possible renal colic. While renal POCUS was negative for hydronephrosis, it demonstrated a complex right renal cystic mass with a combination of anechoic and isoechoic regions separated by multiple thickened septations (Figure 1). POCUS evaluation of the gallbladder, urinary bladder, hepato-renal space, spleno-renal space, and abdominal aorta was unremarkable.

**Figure 2:**
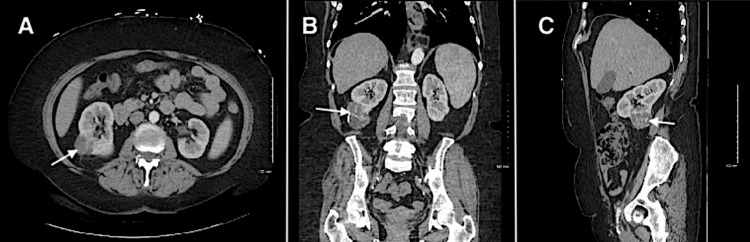
Computed tomography of the abdomen/pelvis demonstrating a large complex Bosniak IV mixed solid-cystic lesion in the right kidney (arrow) in axial (A), coronal (B), and sagittal (C) planes.

Laboratory studies were consistent with the patient’s history of polycythemia vera. There were no electrolyte or renal function abnormalities, and urinalysis was unremarkable for hematuria or underlying urinary infection. CT of the abdomen/pelvis with contrast was obtained to further investigate the abnormal sonographic findings. The CT study was negative for nephrolithiasis or hydronephrosis; however, it demonstrated a 6.3-cm complex Bosniak IV right renal mass with enhancing mixed solid and cystic lesions, highly concerning for malignancy (Figure 2). The patient was ultimately discharged after a urology consultation arranged for an outpatient nephrectomy. Patient follow-up two months later confirmed the diagnosis of renal cell carcinoma (RCC), for which she had undergone surgical resection.

## Discussion

RCC is the ninth and 12th most common malignancy in the United States and worldwide, respectively, comprising approximately 2% of all adult neoplasms [12]. The global incidence of RCC continues to increase particularly in developed countries [12]. Importantly, about 28% of RCCs are diagnosed incidentally with abdominal US or CT, which has likely been attributed to improved survival rates [13]. The diagnostic performance of the Bosniak classification system of CRMs has been reported with sensitivity and specificity up to 90% for CT and sensitivity of 92% and specificity of 91% for MRI [10,11]. CT and MRI are considered the gold-standard imaging modalities for the detailed characterization and staging of renal masses for downstream specialist follow-up; however, there likely is a role for EP-performed POCUS in the early recognition and subsequent management of suspicious renal lesions.

POCUS is an invaluable noninvasive bedside imaging tool for the ED evaluation of a multitude of intra-abdominal and renal pathologies. The POCUS assessment of renal cysts and/or masses has been included in the most updated 2023 Advanced Emergency Ultrasonography Core Content, further emphasizing the need for continued educational training on this specific topic for emergency medicine resident physicians [14]. The Bosniak classification of CRMs has also been developed for ultrasound imaging, with diagnostic features similar to those described for CT and MRI imaging [10]. The diagnostic performance of contrast-enhanced ultrasound (CEUS) for the assessment of CRMs has been reported with a sensitivity of 97.2% and specificity of 71.4% [10]. CEUS, versus non-enhanced ultrasound, allows for a more accurate, real-time differentiation between benign and malignant renal lesions. This accounts for a major limitation of POCUS as was used in this study [10]. Additionally, the diagnostic utility of non-enhanced ultrasound for CRMs has not been previously described. In our patient's case, EPs incidentally detected sonographic features, including irregularly thickened septations with internal anechoic and isoechoic regions representative of fluid-filled and solid areas, respectively, suggestive of a malignant renal mass. We elected to pursue advanced CT imaging in the ED owing to the limitations of non-enhanced POCUS in evaluating CRMs and to further definitively assess for other nonrenal etiologies of flank pain. It is imperative that EPs appropriately document and communicate any actionable incidental findings (AIFs), such as CRMs, determined by POCUS or other advanced imaging [15]. Only 17% of ED patients follow up with AIFs discovered on imaging; thus, EPs are a decisive part of a multidisciplinary team responsible for obtaining time-sensitive diagnostic imaging and specialist consultation [15].

## Conclusions

EP-performed POCUS is an essential initial diagnostic modality to assess for life-threatening etiologies of flank pain in the ED. EPs should also be trained to recognize abnormal renal pathologies, such as CRMs, encountered with POCUS and subsequently initiate the appropriate timely definitive diagnosis and management of these AIFs. Future ED-based studies are certainly warranted to investigate the long-term clinical outcomes of AIFs and POCUS.
